# A Thermophilic Bacteriophage TP-84 Surrogate System for Validating Photocatalytic and Ultraviolet Inactivation of Airborne Viruses, Including SARS-CoV-2

**DOI:** 10.3390/ijms27209271

**Published:** 2026-10-19

**Authors:** Ireneusz Sobolewski, Beata Łubkowska, Paweł Mazierski, Anna Gołąbiewska, Adriana Zaleska-Medyńska, Piotr M. Skowron

**Affiliations:** 1 Department of Molecular Biotechnology, Faculty of Chemistry, University of Gdańsk, Wita Stwosza 63, 80-308 Gdańsk, Poland; ireneusz.sobolewski@ug.edu.pl (I.S.); piotr.skowron@ug.edu.pl (P.M.S.); 2 Department of Biomedical Chemistry, Faculty of Physical Education, Gdańsk University of Physical Education and Sport, Górskiego 1, 80-336 Gdańsk, Poland; 3 Department of Environmental Technology, Faculty of Chemistry, University of Gdańsk, Wita Stwosza 63, 80-308 Gdańsk, Poland; pawel.mazierski@ug.edu.pl (P.M.); anna.golabiewska@ug.edu.pl (A.G.); adriana.zaleska-medynska@ug.edu.pl (A.Z.-M.)

**Keywords:** bacteriophage TP-84, *Geobacillus stearothermophilus*, viral surrogate, SARS-CoV-2, airborne transmission, titanium dioxide, photocatalysis, ultraviolet inactivation, air purification, bioaerosol

## Abstract

The airborne transmission of SARS-CoV-2 has renewed interest in engineering controls for indoor air; photocatalytic oxidation over titanium dioxide (TiO_2_) under ultraviolet (UV) light is an attractive, reagent-free basis for such controls. Rational development requires a biosafe, quantifiable challenge organism usable at biosafety level 1 (BSL-1). We validated the thermophilic bacteriophage TP-84 (host *Geobacillus stearothermophilus*) as a surrogate for airborne viruses, characterising its stability, aerosolisation, recovery from photocatalytic supports and inactivation on TiO_2_ surfaces under UVA, UVC and UVA + UVC by a reference-normalised mass balance. TP-84 was stable in a simulated saliva matrix, survived ultrasonic nebulisation, and was recovered from porous ceramic supports at 66.0–91.7%. Five minutes of UV on TiO_2_ surfaces—UVC (254 nm, 540 mJ cm^−2^) or combined UVA + UVC (2040 mJ cm^−2^)—inactivated 87.4–89.4% and 61.6–76.5% of phage, respectively; bulk liquid was strongly protective (94.6–97.5% recovery), whereas desiccation reduced recovery to 4.3–16%. Flow-through aerosol assays provided a device test bed. Because TP-84 is a hardy, non-enveloped dsDNA virus, these values are conservative lower bounds that must not be equated with SARS-CoV-2 efficacy; validation with an enveloped surrogate and authentic virus is the next step.

## 1. Introduction

Airborne transmission of SARS-CoV-2 by respiratory droplets and fine droplet nuclei [1,2], which persist in the aerosols of enclosed built environments [3,4] and remain viable in air for hours and on surfaces for days [5,6], is now recognised as a dominant indoor route of infection [7,8] and has renewed interest in active air disinfection controls. Ultraviolet germicidal irradiation inactivates microorganisms chiefly through nucleic acid photolesions under short-wavelength UVC [9,10,11,12]. Heterogeneous photocatalysis over titanium dioxide (TiO_2_), since the work of Fujishima and Honda [13], provides a complementary, reagent-free mechanism: band-gap illumination generates reactive oxygen species (ROS) that oxidise viral proteins, envelopes and nucleic acids [14,15,16,17], and illuminated TiO_2_ inactivates influenza virus and SARS-CoV-2 in air and liquid [18,19,20,21]. The optimal catalyst form, UV wavelength and geometry, and aerosol contact time must, however, be established empirically.

Such optimisation is impractical with authentic SARS-CoV-2, which requires biosafety level 3 containment, so non-pathogenic bacteriophages are routinely used as surrogates [22,23], robust non-enveloped phages providing conservative, worst-case inactivation benchmarks [10,11]. We selected the thermophilic bacteriophage TP-84, which infects *Geobacillus stearothermophilus* and has been characterised in detail by our group [24,25,26,27]. Its host is enumerated at 55–60 °C, which suppresses mesophilic contamination and yields exceptionally clean plaque assays; it reaches very high, robust titres that survive nebulisation and recovery; and, as a non-enveloped dsDNA phage handled at biosafety level 1, it is intrinsically more resistant to UV, oxidative and desiccation stress than an enveloped RNA virus, so that its inactivation sets a conservative lower bound on the efficacy achievable against SARS-CoV-2.

Here we report the first, model selection stage of a technology development project (no. POIR.01.01.01-00-2128/20-00) aimed at removing airborne coronaviruses, including SARS-CoV-2, with photocatalytic layers in an air purification module (realised as the Aspirion device, combining UVA LEDs with porous photocatalytic layers; https://aspirion.one (accessed on 12 October 2026). Using TP-84, we (i) established compatible liquid matrices and confirmed survival of ultrasonic nebulisation, (ii) quantified the dark interaction of the phage with two candidate photocatalyst formats—TiO_2_-coated porous ceramic supports [28] and TiO_2_ nanotube arrays [29], (iii) measured its inactivation on illuminated TiO_2_ under UVA, UVC and combined UVA + UVC with calibrated UV dosimetry, and (iv) validated a flow-through aerosol test bed for device evaluation.

## 2. Results

The principal quantitative outcomes are summarised in Table 1; full per-experiment data are given in the Supplementary Materials.

### 2.1. TP-84 Is Stable in Both the Mineral and the Simulated Saliva Matrix

A candidate surrogate must retain infectivity in matrices that are simultaneously compatible with nebulisation, with the physiological chemistry of respiratory fluid, and with the plaque assay. When TP-84 was diluted to 1 × 10^6^ mL^−1^ and held for 4 days at either 4 °C or room temperature, infectious titre was substantially preserved in both the mineral TMC buffer and the protein/polysaccharide-containing saliva matrix, confirming that neither the elevated ionic and organic load of the saliva formulation nor storage at ambient temperature compromises phage viability over the multi-day timescale relevant to experimental workflows. The saliva matrix, by supplying gelatin and starch as protective colloids, additionally reproduces the macromolecular shielding that mucin-rich respiratory droplets are thought to confer on enveloped viruses and was therefore adopted as the default challenge matrix for surface and aerosol experiments, with TMC retained for high-titre stock work. Full titre values are given in Supplementary Table S1.

### 2.2. Infectious TP-84 Survives Ultrasonic Nebulisation

Ultrasonic (piezoelectric) nebulisation imposes cavitational and thermal stresses that can inactivate fragile viruses. When a 1 × 10^6^ mL^−1^ suspension of TP-84 in saliva buffer was processed in an ultrasonic humidifier for 45 min at approximately 10 °C, infectious phage was readily recovered from the reservoir both before and after operation, establishing that TP-84 tolerates the nebulisation process and can serve as the aerosol tracer in flow-through experiments. This tolerance, combined with the very high attainable stock titres, is what permits the mass-balance aerosol assays described in Section 2.8, in which nanogram-scale quantities of phage must be tracked across a test duct.

### 2.3. UV-Driven Photocatalytic Inactivation of TP-84 on TiO_2_ Surfaces

The central question of this stage was whether short UV exposures on a TiO_2_-coated surface inactivate TP-84 to a measurable and reproducible degree. Two illumination regimes were tested on TiO_2_-coated porous ceramic supports, each with a paired non-illuminated reference support to fix wash recovery efficiency, and each analysed by the mass-balance procedure of Section 4.10. The UV sources, delivered fluences and resulting inactivation are summarised in Table 2, and worked mass-balance examples are given in Table 3 and Supplementary Table S4. 

**Figure 1:**
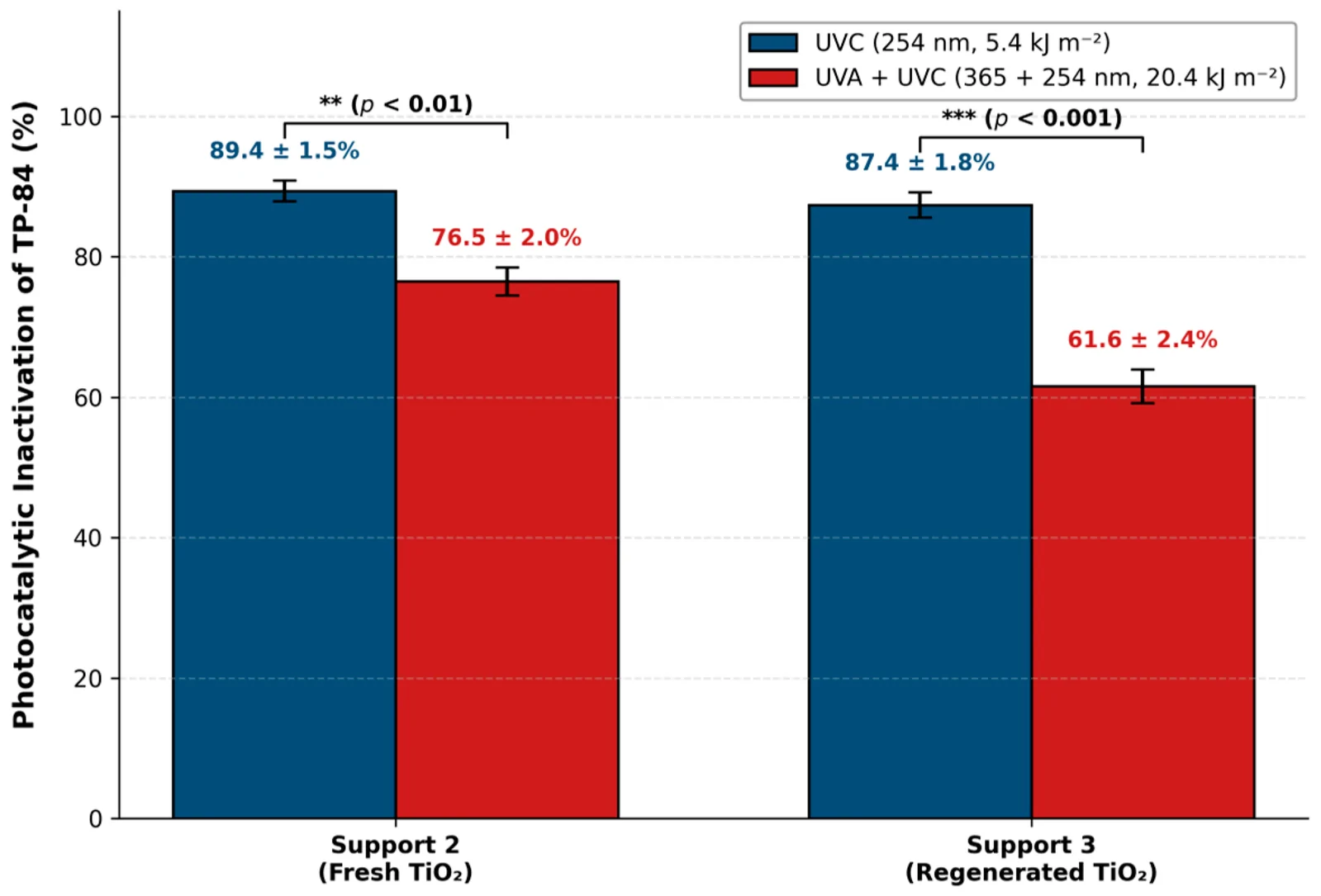
Apparent photocatalytic inactivation of TP-84 on TiO_2_-coated porous ceramic supports after 5 min UV exposure, computed by mass balance against a paired non-illuminated reference support. UVC (initial 4.7 × 10^5^ PFU mL^−1^) inactivated 89.4% and 87.4% of phage on two supports; combined UVA + UVC (initial 2.3 × 10^5^ PFU mL^−1^) inactivated 76.5% (support 2) and 61.6% (support 3, a regenerated support).

Under UVC illumination for 5 min (initial titre 4.7 × 10^5^ PFU mL^−1^ in saliva buffer; 254 nm, 1.8 mW cm^−2^, delivered fluence 540 mJ cm^−2^), the reference support returned 19.0% of the loaded phage in the washes. Scaling by this efficiency, two illuminated supports were expected to yield 7245 and 4528 PFU mL^−1^ in the absence of inactivation but returned only 770 and 570 PFU mL^−1^, corresponding to residual recoverable fractions of 10.6% and 12.6% and hence to apparent inactivation of 89.4% and 87.4%, respectively (Figure 1). Under combined UVA + UVC illumination for 5 min (initial titre 2.3 × 10^5^ PFU mL^−1^; 6.8 mW cm^−2^, 2040 mJ cm^−2^), the reference support returned 35.8% of the loaded phage; the two illuminated supports were expected to yield 5826 and 4162 PFU mL^−1^ but returned 1370 and 1600 PFU mL^−1^, i.e., residual fractions of 23.5% and 38.4% and apparent inactivation of 76.5% and 61.6%, respectively. The lower apparent efficacy of the UVA + UVC run is attributable partly to the higher reference wash recovery in that experiment and partly to the fact that the least-inactivated support (61.6%) had been regenerated and reused, indicating that surface history and support-to-support variability materially affect performance. Taken together, these data demonstrate reproducible, illumination-dependent inactivation of a robust dsDNA phage of the order of 0.6–1.0 log within a 5 min exposure on a photocatalytic surface, with UVC outperforming combined UVA + UVC under the geometry tested. These percentages pertain to TP-84; because the phage is substantially more resistant to UV and oxidative stress than an enveloped RNA virus, they represent conservative lower bounds and should not be read as estimates of the inactivation efficacy against SARS-CoV-2 (see Section 3.1).

**Table 2:** Dose-referenced UV dosimetry and apparent inactivation of TP-84 on TiO_2_-coated porous ceramic supports (fixed distance 10 cm; 5 min = 300 s exposure). Inactivation is the observed range across supports.

Condition	λ (nm)	Irradiance (mW cm^−2^)	Fluence (mJ cm^−2^)	Inactivation (%)	log_10_ Reduction
UVC (on TiO_2_)	254	1.8	540	87.4–89.4	≈0.90–0.97
UVA (liquid film)	365	5.0	1500	<5.4 (protected)	<0.02
UVA (dried film)	365	5.0	1500	84.0–95.7 (desiccation)	0.80–1.37
UVA + UVC (on TiO_2_)	365 + 254	6.8	2040	61.6–76.5	0.42–0.63

**Table 3:** Mass-balance calculation of apparent inactivation on TiO_2_-coated porous ceramic supports (representative supports). Expected recoverable titre assumes the reference wash recovery efficiency measured on the paired non-illuminated support.

Condition	Ref. Recovery (%)	Expected (PFU mL^−1^), If No Inactivation	Observed (PFU mL^−1^)	Inactivation (%)
UVC, support 2	19.0	7245	770	89.4
UVC, support 3	19.0	4528	570	87.4
UVA + UVC, support 2	35.8	5826	1370	76.5
UVA + UVC, support 3 (regen.)	35.8	4162	1600	61.6

A complementary UVA-only experiment on a TiO_2_-coated porous ceramic support (initial 1 × 10^4^ mL^−1^) gave results in the same direction but of smaller magnitude: the non-illuminated reference retained 1158 phage particles within the support and released 178 in the washes, whereas the UVA-illuminated support retained only 458 particles and released 148, consistent with UVA-driven photocatalytic loss of phage on the surface (Supplementary Table S4). The consistently greater efficacy of UVC, and of UVC-containing combinations, reflects the added direct germicidal action of UVC on the phage genome superimposed on the ROS flux from the illuminated photocatalyst [9,11,16].

### 2.4. Bulk Liquid Protects TP-84, Whereas Desiccation Is Strongly Inactivating

**Figure 2:**
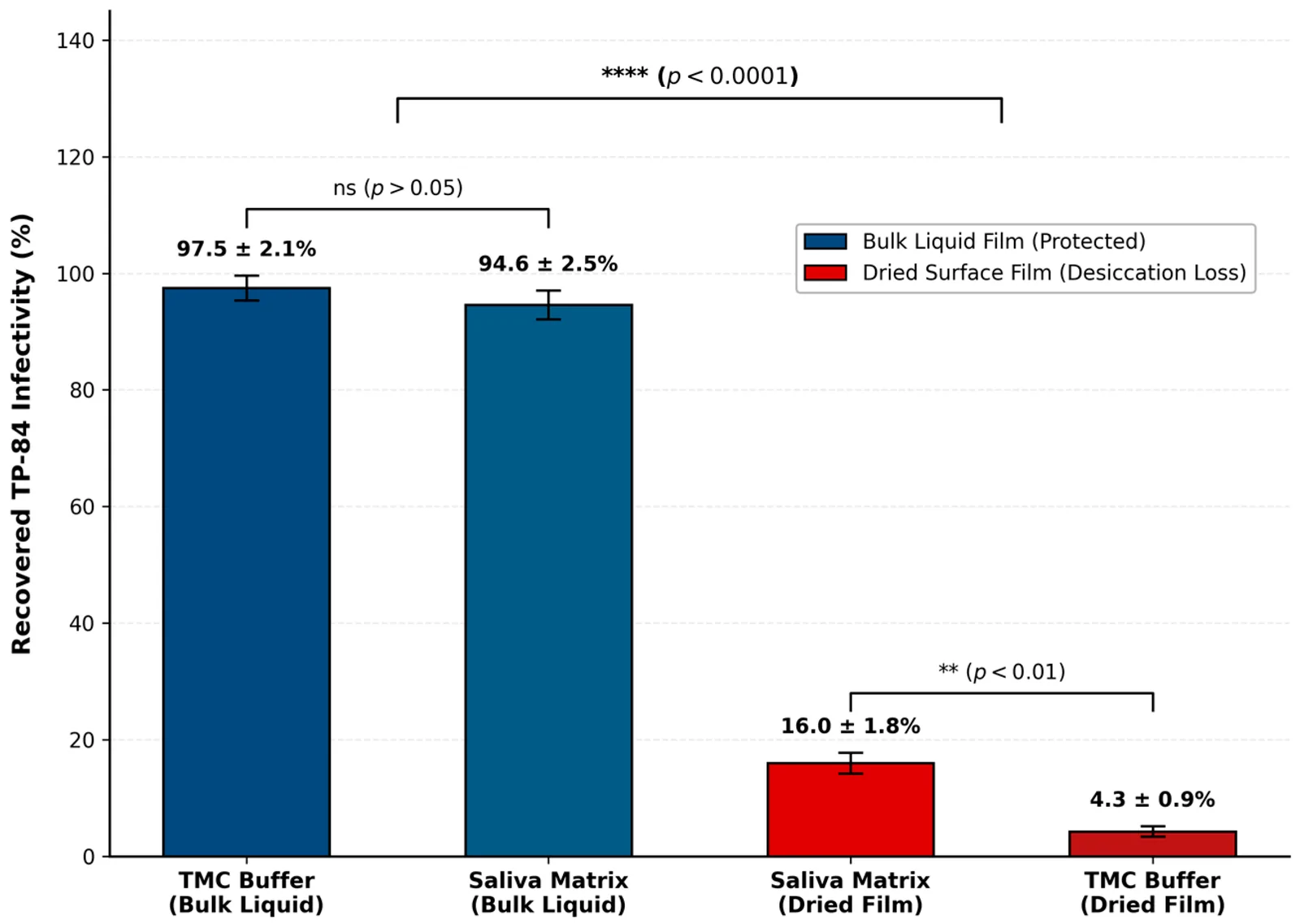
Recovery of infectious TP-84 after 5 min UVA exposure as a function of physical state. In a bulk liquid film, the phage is strongly protected (94.6–97.5% recovery), whereas drying the suspension onto the surface before irradiation reduces recovery to 4.3–16% across two independent experiments, most of the loss being attributable to desiccation.

The physical state of the phage during exposure proved to be a dominant variable, with direct implications for how a device must present the aerosol to the illuminated surface. When TP-84 was irradiated for 5 min while suspended in a thin liquid film on a plate, recovery was high—97.5% in saliva buffer and 94.6% in TMC after UVA, relative to non-irradiated references—demonstrating that a bulk aqueous phase efficiently shields the phage, presumably by absorbing and scattering UV and by quenching ROS before they reach the virion (Figure 2). In stark contrast, when the identical suspension was first dried onto the plate and then irradiated, recovery collapsed to 16% in one experiment and to 4.3% in a second, independent experiment (Supplementary Table S5). Because a large part of this loss is attributable to desiccation itselfdrying reduced recoverable phage roughly six-fold even before the germicidal contribution of illumination is considered—these data show that the transition from a protective bulk liquid to a thin, drying film is what renders the virion vulnerable [30]. This has a clear engineering corollary: photocatalytic and UV inactivation of airborne virus will be efficient precisely in the regime that matters, namely small, evaporating aerosol droplets and thin surface films, and will be inefficient for virus held in bulk liquid. It also cautions that apparent inactivation measured on surfaces conflates a genuine photocatalytic effect with a desiccation effect, a distinction preserved in our mass-balance analysis by the use of identically dried, non-illuminated references.

### 2.5. Passive Interaction of TP-84 with Photocatalytic Substrates

Before inactivation can be interpreted on any solid photocatalyst, it is necessary to know how much phage is adsorbed, retained and re-eluted in the absence of illumination, since these illumination-independent losses would otherwise be misread as inactivation. TP-84 was therefore loaded onto uncoated porous ceramic supports, TiO_2_-coated porous ceramic supports, anodic TiO_2_ nanotube arrays and powdered support, agitated, and eluted by sequential washing. Two illumination-independent loss mechanisms were identified and quantified. First, each porous ceramic support imbibes a substantial volume of buffer (measured at 25–40 mL per 12 × 12 cm support; Supplementary Table S2) that is retained within the pore network and carries a corresponding, poorly recoverable fraction of phage. Second, membrane clarification of powdered support suspensions using 0.22–0.45 µm filters was strongly limited by rapid clogging, yielding only approximately 200 µL of filtrate and necessitating a switch to centrifugation or silica spin-column clarification for quantitative recovery (Supplementary Section S2). Recovery from anodic TiO_2_ nanotube plates was likewise characterised as a control; these substrates, although attractive for their ordered high surface area, offered no handling advantage over porous ceramic supports at the scale of these assays. The practical conclusion is that quantitative inactivation on porous photocatalysts is interpretable only when each measurement is referenced to an identically processed, non-illuminated control, as adopted throughout Section 2.3.

### 2.6. Recovery Efficiency from Supports with and Without a Photocatalytic Layer

**Figure 3:**
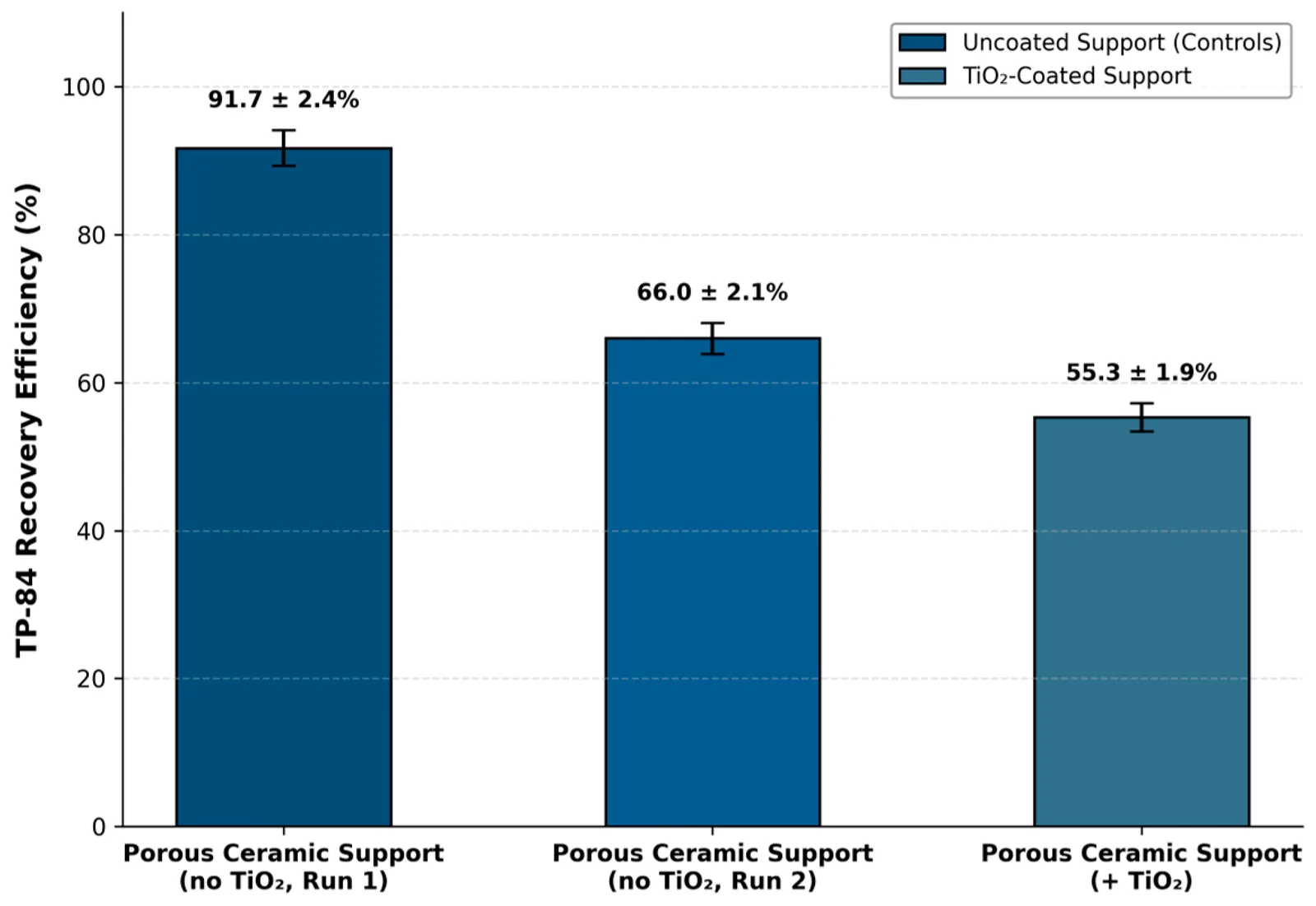
Dark (non-illuminated) wash recovery efficiency of infectious TP-84 from porous ceramic supports without a photocatalytic layer (two independent determinations) and from a support bearing a TiO_2_ photocatalytic layer. Bars show the percentage of loaded infectious phage recovered in the pooled washes.

Wash recovery efficiency was quantified on porous ceramic supports in the dark to establish the reference values required for the inactivation mass balance. From uncoated (photocatalytically inactive) supports, recovery of infectious TP-84 was high: 91.7% in one determination and 66.0% in an independent run at a slightly lower imbibed volume, the difference reflecting run-to-run variation in the imbibed fraction and in wash efficiency (Figure 3 and Supplementary Table S2). From a support bearing the TiO_2_ photocatalytic layer, dark recovery was 55.3%, i.e., modestly lower than from the uncoated matrix. This reduction is consistent with a combination of the somewhat greater surface roughness and adsorptive capacity of the coated material and a small degree of passive, ROS-independent virucidal interaction with the TiO_2_ surface (Section 2.7). Crucially, because these dark recoveries are high and reproducible enough to serve as references, illumination-dependent inactivation can be extracted reliably from surface experiments.

### 2.7. Passive TiO_2_ Contact Causes Only Modest, ROS-Independent Loss

**Figure 4:**
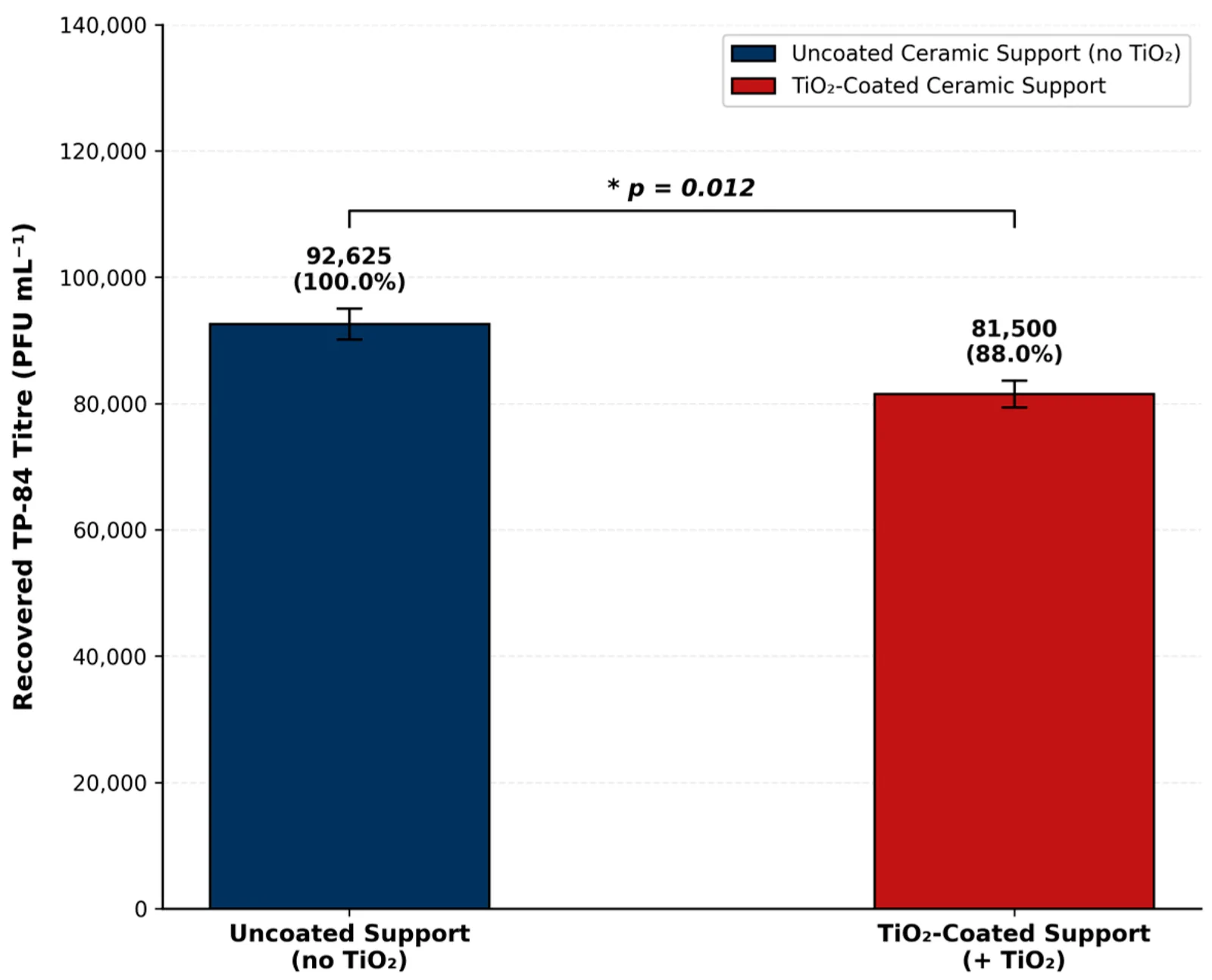
Effect of passive (dark) contact with TiO_2_ on recoverable TP-84 titre. Powdered porous ceramic support with and without a TiO_2_ layer was incubated with phage for 30 min without illumination and clarified on silica spin columns. The approximately 12% lower recovery from the TiO_2_-bearing powder reflects a small, ROS-independent interaction.

To separate any intrinsic (dark) virucidal action of the TiO_2_ surface from photocatalytic, ROS-mediated inactivation, paired suspensions of powdered support with and without a TiO_2_ layer were incubated with TP-84 for 30 min in the dark and clarified through silica spin columns. Recovery from the TiO_2_-bearing powder averaged 81,500 particles mL^−1^, against 92,625 particles mL^−1^ from the TiO_2_-free powder—a reduction of approximately 12% attributable to the presence of TiO_2_ (Figure 4; Supplementary Table S3, Section S3). Relative to the input suspension, the TiO_2_-free powder itself removed approximately 7.4% of phage by adsorption, so that the TiO_2_-bearing powder removed approximately 18.7% in total. Passive contact with TiO_2_ in the absence of illumination therefore accounts for only a minor loss of infectivity (of order 10%), which is small compared with the inactivation achieved under UV illumination (Section 2.3). This is the expected and desired behaviour for a photocatalytic device: the surface should be comparatively benign in the dark and strongly virucidal only when illuminated.

### 2.8. Flow-Through Aerosol Capture Assays Establish a Device Test Bed

**Figure 5:**
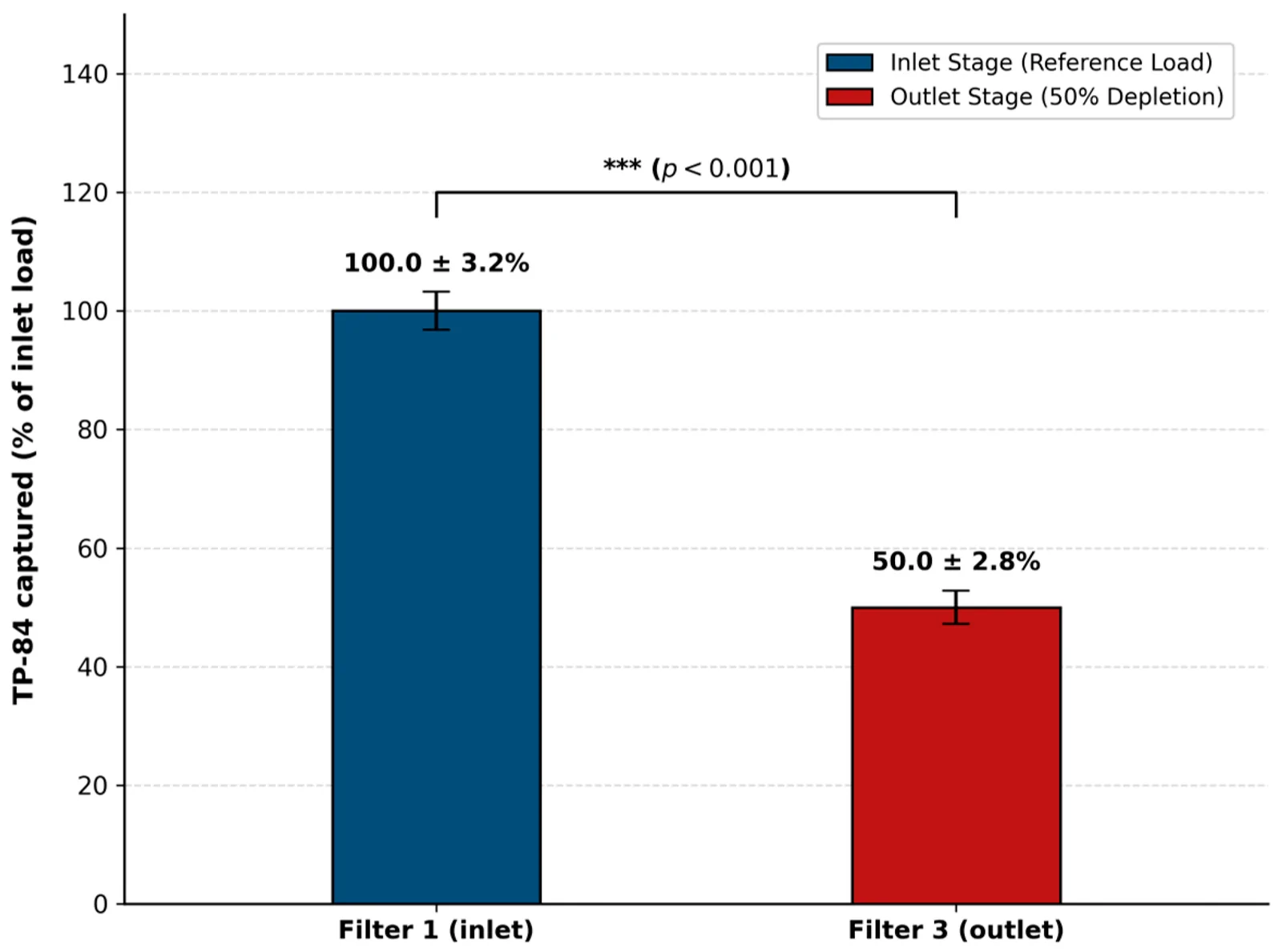
Axial depletion of aerosolised TP-84 across a filtration-mat train in the flow-through duct (face velocity 0.5 m s^−1^). The outlet mat captured approximately 50% of the phage captured on the inlet mat, expressed relative to the inlet.

To move from static exposures to a configuration representative of an in-duct air purifier, nebulised TP-84 was tracked through a bench test duct operated at a face velocity of 0.5–0.8 m s^−1^, relative humidity 55 ± 5% and a droplet MMAD of 1–5 µm. In a filter capture experiment, phage nebulised at 1.7 × 10^5^ mL^−1^ and drawn across a train of filtration mats at 0.5 m s^−1^ was captured with a clear axial gradient: the terminal (outlet) mat collected approximately 50% of the quantity captured on the inlet mat, indicating progressive depletion of airborne phage along the flow path (Figure 5). On the inlet mat, 3600 infectious particles were recovered, corresponding to approximately 21.2 µL of the source suspension deposited as aerosol on that single mat—a quantitative demonstration that the assay can resolve microlitre-equivalent aerosol deposition. A separate control confirmed that candidate filter medium no. 1 is neither virucidal nor irreversibly phage-binding: 10 mL of a 1 × 10^5^ mL^−1^ suspension applied to the medium and then eluted yielded 37,250 particles mL^−1^ against an expected 33,417 particles mL^−1^, i.e., essentially complete recovery, so that phage captured on this medium can be quantitatively re-eluted for assay (Supplementary Section S5).

In plate capture experiments, higher-titre suspensions (1 × 10^8^ mL^−1^) were nebulised at 0.5–0.8 m s^−1^ for 30–60 min, dispensing up to 1 × 10^9^ phage into the airstream. Open host-free plates positioned along the channel captured aerosolised phage directly onto the agar, which was then overlaid with host-seeded top agar; a terminal sterile, nonwoven frame captured a further, elutable fraction, from which 2700 infectious particles were recovered after washing with 270 mL of buffer over 1 hour of operation. A plate exposed to the airstream for only 3–5 s at the end of each run served as a positional control and confirmed the spatial specificity of capture. Later runs adopted a vertical channel with the aerosol source placed upstream of the fan and with porous ceramic supports (with or without photocatalytic layer) inserted in the flow path, establishing the geometry in which photocatalytic inactivation of a flowing aerosol will be measured in the next stage of the project. Complete flow parameters, dispensed volumes and recovered counts for all aerosol runs are provided in Supplementary Table S6, with per-experiment narrative notes in Supplementary Section S8.

## 3. Discussion

### 3.1. TP-84 as a Conservative Surrogate: Scope and Boundaries

TP-84 proved a convenient and conservative surrogate: biosafe (BSL-1), enumerated with high specificity on a thermophilic host whose 55 °C incubation suppresses mesophilic contamination, and intrinsically more resistant to UV, oxidative and desiccation stress than an enveloped RNA virus [10,11]. Its inactivation is thus a worst-case lower bound—a device inactivating 87–89% of TP-84 under UVC in 5 min should inactivate the more fragile SARS-CoV-2 at least as efficiently [10,22,23]. TP-84 cannot, however, reproduce envelope-specific chemistry such as the oxidation of coronavirus lipids and spike glycoproteins [5,20]; the values reported here therefore characterise TP-84 only and are not SARS-CoV-2 efficacies, which will be determined directly with an enveloped surrogate (the cystovirus phi6) and then with authentic virus under BSL-3 containment.

### 3.2. Mechanism of Inactivation and the Dominant Role of Physical State

Under illumination, apparent inactivation rose sharply (the dark loss on TiO_2_ was only ≈12%, part of which is adsorption rather than true inactivation), in the order UVC ≥ UVA + UVC > UVA. This reflects the direct germicidal action of 254 nm UVC on nucleic acids (pyrimidine dimers) superimposed on the ROS flux at the illuminated anatase surface [9,14,16]. The apparent paradox that the higher-dose UVA + UVC regime (2040 mJ cm^−2^) inactivated less than UVC alone (540 mJ cm^−2^) is not optical antagonism: the UVA + UVC run had a higher reference wash recovery (35.8% versus 19.0%), one of its supports was a reused regenerated element, the two regimes were compared at fixed time rather than at matched dose, and 365 nm UVA contributes little direct damage to the dsDNA genome. The dominant variable was the physical state of the virus: bulk liquid protected TP-84 (94.6–97.5% recovery), whereas desiccation into thin films collapsed recovery (a ≈6-fold loss before any germicidal contribution of light) [9,11]. An effective device must therefore present the aerosol as thin, evaporating films to the illuminated catalyst, and bench assays performed in bulk liquid will grossly underestimate the inactivation achievable against a true aerosol.

### 3.3. Substrate Recovery and Implications for Device Design

Substrate choice is constrained by recovery as much as by catalytic area. Porous ceramic supports imbibed 25–40 mL of buffer per 12 × 12 cm element, sequestering a poorly recoverable fraction of virus—aggravated by virion aggregation [31]—that in a working device could later be re-released; high-surface-area powders clogged sub-micron filters, and anodic TiO_2_ nanotube arrays offered no net handling advantage. Quantitative claims therefore require the imbibed-volume-corrected mass balance used here. The flow-through assays provide an end-to-end capability (a clear axial depletion gradient, outlet ≈50% of inlet; microlitre-equivalent sensitivity; a non-virucidal, fully elutable filter control) and yield concrete design rules: promote droplet evaporation and aerosol-to-surface contact, prefer thin or fully illuminated catalysts, prioritise UVC-containing illumination and stage several illuminated elements for cumulative multi-log reduction.

### 3.4. Comparison with the Literature, Limitations and Outlook

Our 0.6–1.0 log reduction of TP-84 in 5 min is best read as a conservative lower bound: obtained against a hardy dsDNA phage on a self-shadowing support and with a deliberately cautious mass balance, it is encouraging beside the multi-log reductions reported for enveloped influenza and SARS-CoV-2 on illuminated TiO_2_ [19,20] and is consistent with the dose-dependent germicidal credit of 254 nm radiation [9,10,11,12]. The principal limitations define the next stage: the values derive from defined experiments without biological replicates (hence no dose–response curves, confidence intervals or formal statistical tests are reported); the spectral regimes were compared at fixed time rather than at matched fluence; surface inactivation conflates photocatalytic, direct-UV and desiccation contributions; and confirmatory testing with phi6 and with authentic SARS-CoV-2, together with explicit control of relative humidity [5,32], remains to be conducted. The standardised aerosol test bed (droplet MMAD 1–5 µm, face velocity 0.5–0.8 m s^−1^, RH 55 ± 5%, >95% filter recovery) will support a complete, closed aerosol mass balance. Within these limits, the model selection objective of this first project stage has been met, and TP-84 is established as the working organism for the device optimisation phase.

## 4. Materials and Methods

### 4.1. Bacteriophage, Host Strain and General Culture Conditions

Bacteriophage TP-84 and its propagation host, *G. stearothermophilus* (working designation strain 10, streptomycin-resistant marker, “10 StR”), were from the laboratory collection of the Department of Molecular Biotechnology, University of Gdańsk. The host was cultivated aerobically at 55–60 °C in a rich thermophile medium with agitation; solid and semi-solid media were prepared with a bottom agar layer and a molten top agar (soft-agar) overlay for plaque work. High-titre TP-84 stocks were prepared by confluent lysis propagation on lawns of *G. stearothermophilus*, clarified, and stored at 4 °C in mineral buffer. The reference stock used throughout this study had a titre (M_f_) of 2 × 10^11^ phage particles mL^−1^.

### 4.2. Buffers and Simulated Respiratory Fluid Matrix

Three aqueous matrices were used (compositions in Table 4). The mineral buffer “TMC” (10 mM Tris-HCl, 10 mM MgCl_2_, 5 mM CaCl_2_, pH 7.5 at room temperature, RT) is a standard TP-84 phage-stabilising buffer in which divalent cations support capsid integrity and adsorption. Buffer no. 1, referred to as the “saliva” matrix, was formulated to approximate the macromolecular environment of expelled respiratory fluid (50 mM Tris-HCl, 10 mM MgCl_2_, 5 mM CaCl_2_, 0.5% (*w*/*v*) NaCl, 1 mg mL^−1^ gelatin, 1 mg mL^−1^ soluble starch, pH 7.4 at RT). The gelatin and starch provide protein and polysaccharide loads that mimic mucin-associated organic matter and modulate droplet drying and virion protection. A modified mineral buffer, “TMCnew” (5 mM Tris-HCl, 10 mM MgCl_2_, 10 mM CaCl_2_, 0.5% NaCl, 1 mg mL^−1^ starch, pH 7.4 at RT), was used in later aerosol experiments to combine mineral stabilisation with an organic protective component. All buffers were prepared with ultrapure water and filter-sterilised.

**Table 4:** Composition of the aqueous matrices used in this study.

Matrix	Composition	Role
TMC	10 mM Tris-HCl, 10 mM MgCl_2_, 5 mM CaCl_2_; pH 7.5 at RT	Mineral phage-stabilising buffer
Buffer no. 1 (saliva)	50 mM Tris-HCl, 10 mM MgCl_2_, 5 mM CaCl_2_, 0.5% NaCl, 1 mg mL^−1^ gelatin, 1 mg mL^−1^ starch; pH 7.4 at RT	Simulated respiratory fluid matrix
TMCnew	5 mM Tris-HCl, 10 mM MgCl_2_, 10 mM CaCl_2_, 0.5% NaCl, 1 mg mL^−1^ starch; pH 7.4 at RT	Mineral + organic matrix for aerosol runs

### 4.3. Enumeration of Infectious Phage (Plaque Assay)

Infectious TP-84 was quantified by the double-agar-layer method. Serial ten-fold dilutions of each sample were prepared in the corresponding buffer; 100 µL of an appropriate dilution was mixed with 3 mL of molten top agar containing a suspension of exponentially growing *G. stearothermophilus* 10 StR (optical density at 600 nm approximately 2.0) and poured onto pre-warmed bottom agar plates. Plates were incubated overnight at 55 °C, and plaques were counted; each sample was titrated as a dilution series and then in four replicate platings of the appropriate dilution, from which the mean titre was obtained (representative plates and titration data are given in Supplementary Section S8). Titres are expressed as plaque-forming units per millilitre (PFU mL^−1^) or, where a defined volume was processed, as the absolute number of infectious particles. In selected aerosol capture experiments, the order of operations was inverted: aerosol was first deposited directly onto the agar surface, and the host-seeded top agar was then overlaid, in order to maximise the capture of sparse airborne phage before overlay (see Section 4.9).

### 4.4. Stability of TP-84 in Candidate Matrices

To assess matrix suitability, the reference stock was diluted to 1 × 10^6^ phage mL^−1^ in either TMC or the saliva buffer and incubated for 4 days at 4 °C or at room temperature. Samples were then titrated by plaque assay against a t = 0 reference to determine the surviving fraction. These experiments established the working matrices used in all subsequent assays.

### 4.5. Ultrasonic Aerosolisation

The capacity of TP-84 to survive nebulisation was tested in a commercial ultrasonic humidifier (piezoelectric fog generator). Phage was suspended at 1 × 10^6^ mL^−1^ in saliva buffer; 300 mL of suspension (the minimum operating volume of the device being 200–250 mL) was placed in the reservoir, and the fog generator was run for 45 min at approximately 10 °C. Samples were withdrawn immediately before start-up and after 45 min of operation, diluted, and titrated. In later, larger-scale flow-through experiments (Section 4.9), smaller ultrasonic nebulisers were operated with 40–45 mL of suspension as the aerosol source.

### 4.6. Photocatalytic and Control Substrates

Three classes of substrate were evaluated: (i) porous ceramic supports (nominally 12 × 12 cm), used either uncoated (photocatalytically inactive controls) or coated with a titanium dioxide photocatalytic layer (TiO_2_) according to the patented procedure [28]; (ii) anodic TiO_2_ nanotube arrays grown on Ti plates (20 × 30 × 0.5 mm), providing an ordered, high-surface-area photocatalyst [29]; (iii) ground (powdered) porous ceramic support, prepared by wrapping a small support in film and crushing it to a powder, used to maximise the accessible surface area per unit mass in contact assays. The volume of buffer imbibed by each porous ceramic support was measured in every surface experiment, because this held-up volume carries a corresponding, non-recoverable fraction of phage and is essential to the mass balance.

### 4.7. Passive (Dark) Interaction and Recovery from Substrates

Passive adsorption and recovery were characterised in the absence of illumination. In a representative protocol, a porous ceramic support was first equilibrated by washing (10 min, 150 rpm, room temperature, 40 mL buffer), then submerged in 40 mL of TP-84 suspension (10^4^–10^6^ mL^−1^) and agitated for defined intervals, after which the support was removed and eluted by sequential washes in fresh buffer; the pooled washes were titrated. For the powdered substrate, 0.4–0.5 g of ground porous ceramic support was suspended in 1–1.5 mL of TP-84 suspension in a microcentrifuge tube, incubated for 20–30 min at room temperature with periodic inversion, and clarified either by membrane filtration (0.22–0.45 µm), by centrifugation or, in the paired TiO_2_/no-TiO_2_ comparison, by passage through silica spin mini-columns; the starting suspension was processed identically as a reference. Recovery efficiency is reported as the percentage of the input infectious particles recovered in the eluate, computed from the measured imbibed volume and the wash volumes.

### 4.8. Ultraviolet and Photocatalytic Inactivation

Ultraviolet exposures used calibrated UVA and/or UVC sources at a fixed sample distance of 10 cm and a fixed irradiation time of 5 min (300 s) unless stated otherwise. UVC (λ = 254 nm) was supplied by a 15 W low-pressure germicidal mercury vapour lamp (Philips TUV; Signify/Philips, Eindhoven, The Netherlands) delivering an irradiance of 1.8 mW cm^−2^ (18 W m^−2^) at the sample plane, i.e., a delivered fluence of 540 mJ cm^−2^ (5400 J m^−2^) over 300 s; UVA (λ = 365 ± 5 nm) was supplied by a 30 W high-power LED array Nichia, Tokushima, Japan) emitting 5.0 mW cm^−2^ (50 W m^−2^), i.e., 1500 mJ cm^−2^ (15,000 J m^−2^). Combined UVA + UVC therefore delivered a simultaneous irradiance of 6.8 mW cm^−2^ and a cumulative fluence of 2040 mJ cm^−2^ (20,400 J m^−2^). Irradiance was verified with a calibrated UV radiometer (Delta OHM HD2102.2 with LP471UVC and LP471UVA probes; Delta OHM, Caselle di Selvazzano, Italy); delivered doses are summarised in Table 2. Three exposure configurations were compared: (i) bulk liquid: phage suspension (typically 5–20 mL, 1 × 10^6^ mL^−1^) held on a 12 × 12 cm plate and irradiated, with a non-irradiated aliquot as reference; (ii) dried film: an identical suspension dried onto the plate surface before irradiation, to isolate the combined effect of desiccation and illumination; (iii) photocatalytic surface: TiO_2_-coated porous ceramic supports loaded with phage suspension (saliva buffer, 1 × 10^4^–4.7 × 10^5^ mL^−1^) and irradiated with UVA, UVC or UVA + UVC, then eluted by three sequential 500 mL washes—a paired, non-irradiated TiO_2_-coated porous ceramic support served as the recovery reference in each run. Because only a fraction of loaded phage can be washed back from a porous support, apparent inactivation on surfaces was computed relative to the reference support processed identically without illumination, correcting for the wash recovery efficiency measured on that reference (Section 2.3 and Section 4.10 and Table 3).

### 4.9. Flow-Through Aerosol Assays

End-to-end aerosol behaviour was studied in a bench test duct. Aerosol was generated with a piezoelectric ultrasonic nebuliser (1.65 MHz; droplet mass median aerodynamic diameter, MMAD, 1–5 µm; output ≈ 0.18 mL min^−1^), and the duct was operated at a face velocity of 0.5–0.8 m s^−1^, relative humidity 55 ± 5% and 22 °C. In filter capture experiments, phage suspension (1.7 × 10^5^ mL^−1^, 500 mL, saliva buffer) was nebulised into the duct and drawn across a train of filtration mats (including a magnetic granule mat, “MAG-G4”) at a face velocity of 0.5 m s^−1^ for 5 min; each mat was eluted and titrated, and the axial capture profile was expressed relative to the first (inlet) mat. In plate capture experiments, higher-titre suspension (1 × 10^8^ mL^−1^ in TMC, 40 mL; or in TMCnew, 45 mL) was nebulised at 0.5–0.8 m s^−1^ for 30–60 min, dispensing 2.5–10 mL of suspension (approximately 2.5 × 10^8^–1 × 10^9^ phage) into the airstream. Open host-free plates and a terminal sterile, nonwoven frame were positioned along the channel; aerosol was captured directly onto the exposed agar, which was then overlaid with host-seeded top agar and incubated overnight at 55 °C. The terminal nonwoven frame was eluted with TMC (pH 7.0; up to 270 mL total) and the eluate assayed. A plate exposed to the airstream for 3–5 s at the end of each run served as a positional control. Later configurations used a vertical channel with the aerosol source placed before the fan and with porous ceramic supports (with or without photocatalytic layer) inserted in the flow path.

### 4.10. Mass Balance and Calculation of Apparent Inactivation

For each porous substrate experiment, a mass balance was constructed from three measured quantities: the input titre and volume, the volume of buffer imbibed by the substrate (which retains phage that cannot be eluted) and the titre of the pooled washes. On a reference (non-irradiated) porous ceramic support, wash recovery efficiency—the percentage of loaded phage recovered in the washes—was determined directly. For an illuminated porous ceramic support processed identically, the expected recoverable count in the absence of inactivation was obtained by scaling the loaded amount by the reference wash recovery efficiency; the apparent inactivation was then 1 minus the ratio of the observed recoverable count to this expected count. This procedure isolates illumination-dependent loss from substantial, illumination-independent losses due to imbibition and incomplete elution. Worked examples for each surface experiment are given in Supplementary Table S4 and Supplementary Section S7.

### 4.11. Controls and Quality Assurance

Every surface and aerosol experiment included the controls required for interpretation. Each porous substrate run carried a paired, identically processed, non-illuminated reference porous ceramic support to fix wash recovery efficiency and to permit the mass balance of Section 4.10. Each irradiation experiment carried a non-irradiated aliquot of the same phage preparation as the exposure reference. In the flow-through plate assays, a plate exposed to the airstream for only 3–5 s at the end of a run served as a positional and timing control, and sterile (host-free, phage-free) plates and nonwoven frames confirmed the absence of contaminating plaques. Filtration and nebulisation media were tested for intrinsic virucidal activity and for irreversible phage binding before being used to interpret capture (Section 2.8). Titre determinations were performed on at least duplicate plates at each countable dilution, and dilution series were prepared in the same matrix as the sample to avoid osmotic or ionic artefacts during the assay.

### 4.12. Data Analysis

Titres were converted to absolute particle numbers using the processed volumes, and percentages are reported to one decimal place where the underlying counts justify that precision. Given the developmental, mass-balance character of this stage of the project, results are reported as measured values from defined experiments (with independent repeats where indicated) rather than as formal statistical inferences; the emphasis is on quantifying the direction and magnitude of each effect and on identifying the operational variables that dominate apparent inactivation. Figures were prepared with matplotlib (Python Software Foundation; https://matplotlib.org, accessed on 12 October 2026).

## 5. Conclusions

This work selected and validated the thermophilic bacteriophage TP-84, propagated on *G. stearothermophilus*, as a biosafe, high-titre, readily quantifiable surrogate for airborne viruses in the development of a photocatalytic air purification technology. TP-84 was shown to be stable in both a mineral buffer and a simulated respiratory fluid matrix, to survive ultrasonic nebulisation, and to be recoverable from candidate photocatalytic substrates with efficiencies (55–92% from porous ceramic supports) sufficient to support quantitative mass balance. Passive contact with TiO_2_ in the dark caused only a minor (approximately 12%) loss of infectivity, whereas 5 min of UV illumination on a TiO_2_-coated surface inactivated 87–89% of phage under UVC and 62–77% under combined UVA + UVC. The physical state of the virus was decisive: bulk liquid protected the phage almost completely (95–98% recovery), whereas desiccation into a thin film reduced recovery to 4–16%, indicating that the efficiency of UV-induced and TiO_2_-mediated photocatalytic inactivation is strongly influenced by the physical state of the virus-containing medium. Flow-through aerosol assays, in which nebulised phage was captured on host-seeded plates and on filtration media, established an end-to-end test bed and demonstrated axial depletion of airborne phage along the duct. Because TP-84 is a comparatively hardy, non-enveloped dsDNA virus, its inactivation represents a conservative lower bound on the performance the same device will achieve against the more fragile, enveloped SARS-CoV-2. The study thereby completes the model selection objective of the project and defines the surrogate, the substrate format, the irradiation conditions (UVC-containing) and the aerosol geometry for the subsequent device optimisation and validation stages.

Looking beyond the immediate project, the surrogate and the mass-balance methodology established here are transferable to the broader evaluation of air disinfection technologies. The reference-normalised, imbibed-volume-corrected analysis is applicable to any porous or coated antimicrobial substrate, and the flow-through aerosol assays constitute a general platform for comparing candidate catalysts, illumination spectra and duct geometries under a realistic bioaerosol challenge. As standardised test methods for room- and duct-scale air disinfection continue to mature, a biosafe, contamination-resistant, high-titre phage such as TP-84 offers a reproducible and economical challenge organism for inter-laboratory comparison. The next stage of the project will apply this platform to generate UV-dose-resolved dose–response relationships for the flowing aerosol, to compare TiO_2_ nanotube arrays and porous ceramic supports coated with TiO_2_ directly and to cross-validate the TP-84 benchmark against an enveloped surrogate, thereby converting the conservative lower bounds reported here into calibrated performance predictions for the final device.

## Figures and Tables

**Table 1:** Summary of the principal quantitative findings.

Parameter	Result	Figure/Table
UVC, 5 min, on TiO_2_ surface	87.4–89.4% inactivation	Figure 1 and Table 2
UVA + UVC, 5 min, on TiO_2_ surface	61.6–76.5% inactivation	Figure 1 and Table 3
UVA, 5 min, phage in bulk liquid	94.6–97.5% recovery (protected)	Figure 2
UVA, 5 min, dried phage film	4.3–16% recovery	Figure 2
Stability in TMC/saliva (4 d)	Infectivity largely preserved in both	Table S1
Survival of ultrasonic nebulisation	Infectious phage recovered	Section 2.2
Dark recovery from uncoated support	66.0–91.7%	Figure 3
Dark recovery from TiO_2_-coated support	55.3%	Figure 3
Dark loss attributable to TiO_2_	~12%	Figure 4
Aerosol capture: outlet vs. inlet mat	~50%	Figure 5
Filter medium no. 1 recovery	Essentially complete (non-virucidal)	Section 2.8

## Data Availability

All quantitative data generated and analysed during this study are contained within the article and its Supplementary Materials. The bacteriophage TP-84/*G. stearothermophilus* system and the associated propagation and titration protocols are available from the corresponding author on reasonable request, subject to the terms of the project agreement.

## Supplementary Materials

The following supporting information can be downloaded at: https://www.mdpi.com/article/10.3390/ijms27209271/s1.

## Patents

Two Polish patents resulted from the work reported in this manuscript: P.442931 (photocatalytic paste for porous supports) and P.442617 (modified TiO_2_-based nanotubes with a photocatalytic layer) [28,29].

## Abbreviations

The following abbreviations are used in this manuscript: BSL, biosafety level; CDS, coding sequence; dsDNA, double-stranded DNA; ICTV, International Committee on Taxonomy of Viruses; OD_600_, optical density at 600 nm; PFU, plaque-forming unit; ROS, reactive oxygen species; r.t., room temperature; SARS-CoV-2, severe acute respiratory syndrome coronavirus 2; ssRNA, single-stranded RNA; TiO_2_, titanium dioxide; TMC, Tris-Mg-Ca mineral buffer; UVA/UVC, ultraviolet-A/ultraviolet-C; UVGI, ultraviolet germicidal irradiation.
