# Supplementary material for: A Thermophilic Bacteriophage TP-84 Surrogate System for Validating Photocatalytic and Ultraviolet Inactivation of Airborne Viruses, Including SARS-CoV-2
Source: Int J Mol Sci. 2026 Oct 19;27(20):9271. doi: 10.3390/ijms27209271 (PMC13705311; doi:10.3390/ijms27209271)
Supplement: Supplementary file 1 [file ijms-27-09271-s001.zip › Supplementary S1.pdf]

Supplementary Material

This Supplementary Material provides the protocol-level detail and the complete, per-experiment data underlying the main text. Section S1 gives the experiment log; Sections S2-S6 give expanded methods and full data tables for stability, substrate interaction, surface inactivation, and aerosol experiments. Supplementary tables are numbered S1-S6.

S1. Experiment log (Stage 1)

Table S1 lists every experiment of Stage 1 with its substrate/condition, input titre and principal quantitative outcome, and serves as an index to the detailed sections that follow.

Table S1. Log of Stage 1 experiments.

No.	Experiment / substrate	Input titre	Principal outcome
1	Stability in TMC vs saliva buffer (4 d, 4 °C / r.t.)	1×10^6 mL^{-1}	Phage stable in both matrices
2	Ultrasonic humidifier stability (45 min, ~10 °C)	1×10^6 mL^{-1}	Infectious phage recovered post-nebulisation
3	Porous ceramic support interaction (wash + contact)	1×10^6 mL^{-1}	Baseline adsorption/elution established
4	Porous ceramic support interaction, low titre	1×10^4 mL^{-1}	3× wash elution protocol
5	TiO_2 nanotube plates	1×10^6 mL^{-1}	Recovery control on nanotubes
6	TiO_2 nanotube plates, low titre	1×10^4 mL^{-1}	Recovery control on nanotubes
7	Ground support; membrane vs centrifugation	1×10^4	0.22/0.45 µm membranes clog; ~200 µL filtrate
8	UVA on TiO_2-coated support, 5 min	1×10^4 mL^{-1}	Ref 178 vs UVA 148 washed; in-support 1158 to 458
9	UVA/UVC of phage in solution	1×10^6 mL^{-1}	Recovery 94.6-97.5 %; dried 16 %
10	UVA/UVC of dried phage	1×10^6 mL^{-1}	Recovery 4.3-6.25 %
11	Recovery from support, no TiO_2 layer	1×10^4 mL^{-1}	91.7 % recovery (40 mL imbibed)
12	Recovery from support, no TiO_2 layer	1×10^4 mL^{-1}	66.0 % recovery (32 mL imbibed)
13	Recovery from support, + TiO_2 layer	1×10^4 mL^{-1}	55.3 % recovery (40 mL imbibed)
14	Effect of TiO_2 on phage (plates, dark)	1×10^4 mL^{-1}	5/30 min dark contact
15	Ground support without TiO_2	1×10^5	Adsorption baseline
16	UVC on support + TiO_2, 5 min	4.7×10^5 mL^{-1}	Inactivation 89.4 % / 87.4 %
17	UVA+UVC on support + TiO_2, 5 min	2.3×10^5 mL^{-1}	Inactivation 76.5 % / 61.6 %
18	Ground support ± TiO_2 (dark, spin columns)	1×10^5 mL^{-1}	+TiO_2: 12 % lower recovery
19	Filter medium no. 1 capture/elution	1×10^5 mL^{-1}	Full recovery; non-virucidal
20	Flow-through MAG-G4 mat train	1.7×10^5 mL^{-1}	Outlet 50 % of inlet capture
21	Flow-through plate capture (TMC)	1×10^8 mL^{-1}	2700 phage on nonwoven / h
22	Flow-through plate capture (TMCnew)	1×10^8 mL^{-1}	Vertical channel, ± supports
