# Supplementary material for: A Thermophilic Bacteriophage TP-84 Surrogate System for Validating Photocatalytic and Ultraviolet Inactivation of Airborne Viruses, Including SARS-CoV-2
Source: Int J Mol Sci. 2026 Oct 19;27(20):9271. doi: 10.3390/ijms27209271 (PMC13705311; doi:10.3390/ijms27209271)
Supplement: Supplementary file 1 [file ijms-27-09271-s001.zip › Supplementary S2.pdf]

S2. Expanded methods: stability, aerosolisation and substrate interaction

S2.1. Matrix stability

TP-84 reference stock ($M_f = 2\times10^{11}$ mL^{-1}) was diluted to 1×10^{6} mL^{-1} in TMC or saliva buffer and incubated for four days at 4 °C or room temperature before plaque titration against a t = 0 control. The saliva matrix (Table 1) was adopted as the default challenge matrix because its gelatin/starch load reproduces the protective organic environment of respiratory fluid while remaining fully compatible with the plaque assay.

S2.2. Ultrasonic nebulisation

Phage at 1×10^{6} mL^{-1} in saliva buffer (300 mL; device minimum operating volume 200-250 mL) was nebulised for 45 min at approximately 10 °C. Aliquots taken before start-up and after 45 min were titrated. Smaller nebulisers (40-45 mL) were used as aerosol sources in the flow-through assays (Section S6).

S2.3. Porous ceramic support interaction protocols

Representative protocol: a porous ceramic support was washed by mixing in 40 mL buffer for 10 min (150 rpm, r.t.), then submerged in 40 mL of TP-84 (1×10^{6} mL^{-1}, saliva) and mixed for 30 min (150 rpm, r.t.) followed by 30 min static incubation; the support was removed and eluted in 40 mL fresh buffer (30 min, 220 rpm). Low-titre protocol: 40 mL of TP-84 at 1×10^{4} mL^{-1}, gentle mixing (60 rpm) for 60 min, followed by three sequential 30 min washes (200 rpm), the support being transferred to a fresh vessel and shaken between washes to expel residual liquid.

S2.4. TiO_2 nanotube arrays

Two small anodic TiO_2 nanotube plates were pre-washed in 10 mL buffer (100 rpm, 10 min), then exposed to 10 mL (1×10^{6} mL^{-1}) or 20 mL (1×10^{4} mL^{-1}) of TP-84 for 1 h (60-100 rpm), a start aliquot being reserved for reference; plates were then eluted by sequential washes in fresh buffer (three washes for the low-titre run).

S2.5. Ground-support clarification

A small porous ceramic support was wrapped in film and crushed to powder. Aliquots of 0.4-0.5 g were suspended in 1-1.5 mL of TP-84 (1×10^{4}-1×10^{5} mL^{-1}, saliva) and incubated 20-30 min at r.t. with periodic inversion. Clarification was attempted by (i) syringe filtration through 0.45 µm (clogged; 200 µL recovered), (ii) centrifugal filtration through 0.22 µm (16 000 × g, 1 h; clogged), or (iii) direct centrifugation (16 000 × g, 3 min) with recovery of 500 µL supernatant. The paired ± TiO_2 comparison used silica spin mini-columns, with the starting suspension processed identically.
