# Supplementary material for: A Thermophilic Bacteriophage TP-84 Surrogate System for Validating Photocatalytic and Ultraviolet Inactivation of Airborne Viruses, Including SARS-CoV-2
Source: Int J Mol Sci. 2026 Oct 19;27(20):9271. doi: 10.3390/ijms27209271 (PMC13705311; doi:10.3390/ijms27209271)
Supplement: Supplementary file 1 [file ijms-27-09271-s001.zip › Supplementary S3.pdf]

S3. Full recovery and imbibition data

Table S2 compiles the imbibed-volume and recovery data for the porous ceramic support experiments summarised in Figure 2, and Table S3 gives the ground-support ± TiO_2 dark-contact data underlying Figure 4.

Table S2. Recovery of TP-84 from porous ceramic supports (dark), with imbibed volumes and derived particle counts.

Run	Layer	Imbibed vol.	In-support particles / PFU mL^{-1}	Recovery
1	No TiO_2	40 mL	344 000 / 688	91.7 %
2	No TiO_2	32 mL	305 056 / 1016	66.0 %
3	+ TiO_2	40 mL	381 320 / 1271	55.3 %

Table S3. Dark contact of TP-84 with ground porous ceramic support ± TiO_2 (silica spin-column clarification; input 1×10^5 mL^{-1}).

Condition	Recovered (particles mL^{-1})	Loss vs input / vs no-TiO_2
Ground support, no TiO_2	92 625	7.4 % vs input
Ground support, + TiO_2	81 500	18.7 % vs input; 12 % vs no-TiO_2
