# Supplementary material for: A Thermophilic Bacteriophage TP-84 Surrogate System for Validating Photocatalytic and Ultraviolet Inactivation of Airborne Viruses, Including SARS-CoV-2
Source: Int J Mol Sci. 2026 Oct 19;27(20):9271. doi: 10.3390/ijms27209271 (PMC13705311; doi:10.3390/ijms27209271)
Supplement: Supplementary file 1 [file ijms-27-09271-s001.zip › Supplementary S4.pdf]

S4. Full surface-inactivation data

Table S4 gives the complete mass-balance data for the UVC and UVA+UVC experiments on TiO_2-coated porous ceramic supports, including the imbibed volumes of each support and the reference wash-recovery efficiency used to compute expected recoverable counts, together with the UVA-only experiment.

Table S4. Mass-balance data for UV/photocatalytic inactivation on TiO_2-coated porous ceramic supports.

Experiment	Imbibed	Ref. recov.	Expected PFU mL^{-1}	Observed	Inact.
UVC 5 min - ref	30 mL	19.0 %	-	5450 (19 %)	-
UVC 5 min - support 2	40 mL	19.0 %	7245	770 (10.6 %)	89.4 %
UVC 5 min - support 3	25 mL	19.0 %	4528	570 (12.6 %)	87.4 %
UVA+UVC - ref	40 mL	35.8 %	-	6660 (35.8 %)	-
UVA+UVC - support 2	35 mL	35.8 %	5826	1370 (23.5 %)	76.5 %
UVA+UVC - support 3 (regenerated)	25 mL	35.8 %	4162	1600 (38.4 %)	61.6 %
UVA 5 min - ref	-	-	1158 in-support	178 washed	-
UVA 5 min - illuminated	-	-	458 in-support	148 washed	-

The two dried-film experiments are summarised in Table S5. In solution, UVA for 5 min reduced recoverable phage only marginally (to 94.6-97.5 % of the non-irradiated reference), whereas drying reduced recovery to 16 % (first experiment) and, in an independent repeat performed at higher elution stringency, to 4.3-6.25 %.

Table S5. Effect of physical state on recovery after 5 min UVA (input 1×10^{6} mL^{-1}, saliva/TMC).

State / matrix	Recovery after UVA	Note
Solution, simulated saliva	97.5 %	Bulk liquid protects phage
Solution, TMC	94.6 %	Bulk liquid protects phage
Dried film (experiment A)	16 %	~6× loss vs solution
Dried film (experiment B)	4.3-6.25 %	Desiccation dominates loss
