# Supplementary material for: A Thermophilic Bacteriophage TP-84 Surrogate System for Validating Photocatalytic and Ultraviolet Inactivation of Airborne Viruses, Including SARS-CoV-2
Source: Int J Mol Sci. 2026 Oct 19;27(20):9271. doi: 10.3390/ijms27209271 (PMC13705311; doi:10.3390/ijms27209271)
Supplement: Supplementary file 1 [file ijms-27-09271-s001.zip › Supplementary S5.pdf]

S5. Filter-capture control

Candidate filter medium no. 1 was tested for virucidal activity and irreversible binding. Ten millilitres of TP-84 (1×10^5 mL^{-1}, saliva) were applied to the medium; after 30 min the medium was placed in a 50 mL tube and centrifuged (5 000 × g, 3 min), then washed, the eluates being pooled to a final 30 mL. The expected diluted titre was 1 002 500 particles / 30 mL = 33 417 mL^{-1}; the measured titre after elution was 37 250 mL^{-1}, i.e. essentially complete recovery, establishing that the medium neither inactivates nor irreversibly binds the phage and that captured phage can be quantitatively eluted for assay.
