# Supplementary material for: A Thermophilic Bacteriophage TP-84 Surrogate System for Validating Photocatalytic and Ultraviolet Inactivation of Airborne Viruses, Including SARS-CoV-2
Source: Int J Mol Sci. 2026 Oct 19;27(20):9271. doi: 10.3390/ijms27209271 (PMC13705311; doi:10.3390/ijms27209271)
Supplement: Supplementary file 1 [file ijms-27-09271-s001.zip › Supplementary S6.pdf]

S6. Flow-through aerosol assays: full parameters

Table S6 compiles the operating parameters and outcomes of the flow-through aerosol experiments. In the mat-train experiment, capture on the outlet mat was approximately 50 % of that on the inlet mat, and 3 600 particles on the inlet mat corresponded to approximately 21.2 µL of source suspension deposited as aerosol (3 600 / 1.7×10^5 mL^{-1} = 0.0212 mL). In plate-capture experiments, aerosol was deposited directly onto exposed agar subsequently overlaid with host-seeded top agar (OD_{600} approximately 2.0) and incubated overnight at 55 °C; a terminal sterile nonwoven captured 2 700 elutable particles over 1 h. A plate exposed to the airstream for 3-5 s at the end of each run served as a positional control.

Table S6. Flow-through aerosol experiments: parameters and outcomes.

Assay	Input titre	Flow / time	Dispensed	Outcome
MAG-G4 mat train	1.7×10^5 mL^{-1}	0.5 m s^{-1} / 5 min	500 mL nebulised	Outlet 50 % of inlet; 3600 on inlet
Plate capture (TMC)	1×10^8 mL^{-1}	0.8 m s^{-1} / 30-60 min	~1×10^9 phage	2700 phage on nonwoven / h
Plate capture (TMCnew)	1×10^8 mL^{-1}	0.5 m s^{-1} / 45 min (3×15)	2.5×10^8 phage	Vertical channel, ± supports / ± TiO_2
