# Supplementary material for: A Thermophilic Bacteriophage TP-84 Surrogate System for Validating Photocatalytic and Ultraviolet Inactivation of Airborne Viruses, Including SARS-CoV-2
Source: Int J Mol Sci. 2026 Oct 19;27(20):9271. doi: 10.3390/ijms27209271 (PMC13705311; doi:10.3390/ijms27209271)
Supplement: Supplementary file 1 [file ijms-27-09271-s001.zip › Supplementary S7.pdf]

S7. Worked mass-balance calculations

This section sets out, step by step, the arithmetic underlying the apparent-inactivation values reported in the main text, so that the calculations can be reproduced and audited. The general scheme is: (1) determine, on a non-illuminated reference support, the fraction of loaded phage that is recoverable in the pooled washes (the wash-recovery efficiency, R_{ref}); (2) for each illuminated support, compute the count that would be recovered if no inactivation had occurred, as the loaded count scaled by R_{ref} (the expected count, E); (3) compare the observed recovered count, O, with E; and (4) report apparent inactivation as $I = (1 - O/E) \times 100$ %.

S7.1. UVC experiment

Initial titre 4.7×10^5 PFU mL^{-1} (measured 476 667 PFU mL^{-1}), saliva buffer; two illuminated supports plus one reference, each irradiated (or, for the reference, sham-irradiated) for 5 min. The reference support released 19.0 % of its loaded phage into the washes (5 450 PFU mL^{-1}), defining $R_{ref} = 0.190$. For illuminated support 2, the expected recoverable titre was E = 7 245 PFU mL^{-1}; the observed titre was O = 770 PFU mL^{-1} (10.6 % of loaded), giving I = (1 - 770/7 245) × 100 % = 89.4 %. For support 3, E = 4 528 PFU mL^{-1} and O = 570 PFU mL^{-1} (12.6 %), giving I = (1 - 570/4 528) × 100 % = 87.4 %.

S7.2. UVA+UVC experiment

Initial titre 2.3×10^5 PFU mL^{-1} (measured 232 500 PFU mL^{-1}), saliva buffer; combined UVA+UVC, 5 min. The reference support released 35.8 % of its loaded phage (6 660 PFU mL^{-1}), so $R_{ref} = 0.358$. For illuminated support 2, E = 5 826 PFU mL^{-1} and O = 1 370 PFU mL^{-1} (23.5 %), giving I = (1 - 1 370/5 826) × 100 % = 76.5 %. For support 3 (a regenerated, reused support), E = 4 162 PFU mL^{-1} and O = 1 600 PFU mL^{-1} (38.4 %), giving I = (1 - 1 600/4 162) × 100 % = 61.6 %. The higher R_{ref} in this run relative to the UVC run (0.358 vs 0.190) reflects the larger imbibed volumes and more efficient elution, and is precisely the kind of run-to-run variation that the reference-normalised mass balance is designed to absorb.

S7.3. Recovery from supports (Figure 2)

For the first uncoated support, the support imbibed 40 mL, corresponding to 344 000 particles held within the support (equivalent to 688 PFU mL^{-1} when distributed in the 500 mL wash volume); recovery in the washes was 91.7 % of the loaded phage. For the second uncoated support, the imbibed volume was 32 mL (305 056 particles; 1 016 PFU mL^{-1} in 300 mL wash) with 66.0 % recovery. For the TiO_2-coated support, the imbibed volume was 40 mL (381 320 particles; 1 271 PFU mL^{-1} in 300 mL wash) with 55.3 % recovery. The lower recovery from the coated support is attributed to a combination of adsorptive retention and a small dark virucidal interaction (Section 3.7).

S7.4. Aerosol deposition equivalent (Figure 5)

In the MAG-G4 mat-train experiment, the inlet mat captured 3 600 infectious particles. Given a source titre of 1.7×10^5 PFU mL^{-1}, this corresponds to a deposited liquid-equivalent volume of 3 600 / (1.7×10^5 mL^{-1}) = 0.0212 mL, i.e. approximately 21.2 µL of source suspension deposited as aerosol on that single mat. The outlet (third) mat captured approximately 50 % of the inlet quantity, quantifying the axial depletion of airborne phage across the train.
