# Supplementary material for: A Thermophilic Bacteriophage TP-84 Surrogate System for Validating Photocatalytic and Ultraviolet Inactivation of Airborne Viruses, Including SARS-CoV-2
Source: Int J Mol Sci. 2026 Oct 19;27(20):9271. doi: 10.3390/ijms27209271 (PMC13705311; doi:10.3390/ijms27209271)
Supplement: Supplementary file 1 [file ijms-27-09271-s001.zip › Supplementary_S8.pdf]

Supplementary Materials

A Thermophilic Bacteriophage TP-84 Surrogate System for Validating Photocatalytic and Ultraviolet Inactivation of Airborne Viruses, Including SARS-CoV-2

Section S8. Representative Plaque-Assay Titration of Bacteriophage TP-84

Microbiological tests were developed, based on titration on the host *Geobacillus stearothermophilus* 10 StR by the double-agar-layer method, of live bacteriophage TP-84, in order to determine the efficacy of the photocatalytic layers. Each sample was titrated twice: first as a dilution series, to estimate the approximate number of live bacteriophage, and second as four replicate platings of the appropriate dilution, to calculate the mean and determine the live-bacteriophage count precisely. Every experiment involving live bacteriophage reported in the main text concludes with such a microbiological test.

The data below are representative titrations from a single experimental session: interaction of bacteriophage TP-84 with photocatalytic surfaces (ground porous ceramic support ± TiO_2). Preparations are labelled: "Start Sal 10^5" (input phage in simulated-saliva buffer, 1×10^5 PFU mL^{-1}); "– TiO_2 1/2" and "+ TiO_2 3/4" (ground support without and with the TiO_2 photocatalytic layer, respectively); and "F (filter)" (eluate from the candidate filter medium). Each cell gives the plaque count and the corresponding titre (plaques / PFU mL^{-1}).

Table S8.1. First titration (dilution series; experimental session of 23–24 February 2022). Values are plaques / PFU mL^{-1}.

Preparation	Dilution 10^{-1} [plaques / PFU]	Dilution 10^{-2} [plaques / PFU]	Dilution 10^{-3} [plaques / PFU]
Start Sal 10^5	350 / 3.5×10^4	120 / 1.2×10^5	15 / 1.5×10^5
– TiO_2 1	300 / 3.0×10^4	97 / 9.7×10^4	6 / 6.0×10^4
– TiO_2 2	300 / 3.0×10^4	69 / 6.9×10^4	14 / 1.4×10^5
+ TiO_2 3	290 / 2.9×10^4	114 / 1.1×10^5	9 / 9.0×10^4
+ TiO_2 4	250 / 2.5×10^4	69 / 6.9×10^4	10 / 1.0×10^5
F (filter)	200 / 2.0×10^4	55 / 5.5×10^4	7 / 7.0×10^4

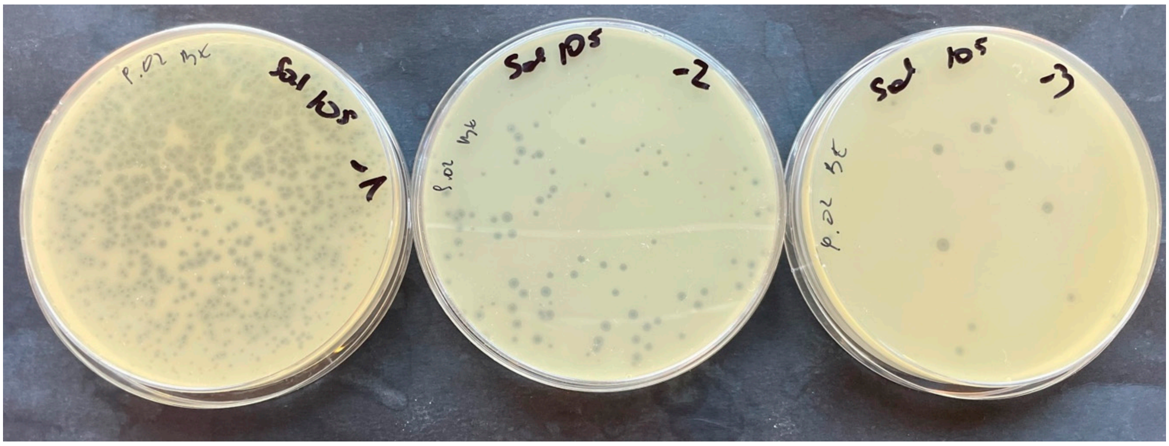

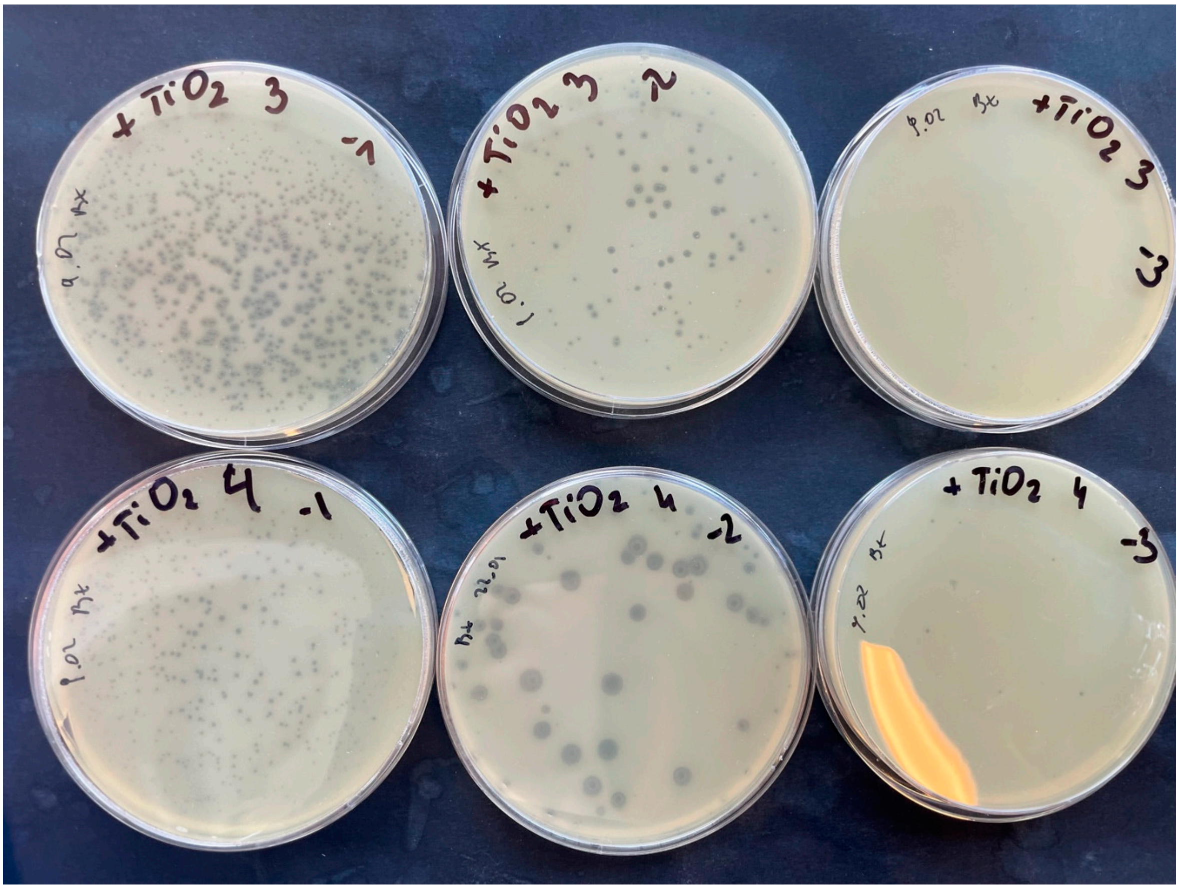
+TiO2 3 -1
+TiO2 3 -2
+TiO2 3 -3
+TiO2 4 -1
+TiO2 4 -2
+TiO2 4 -3

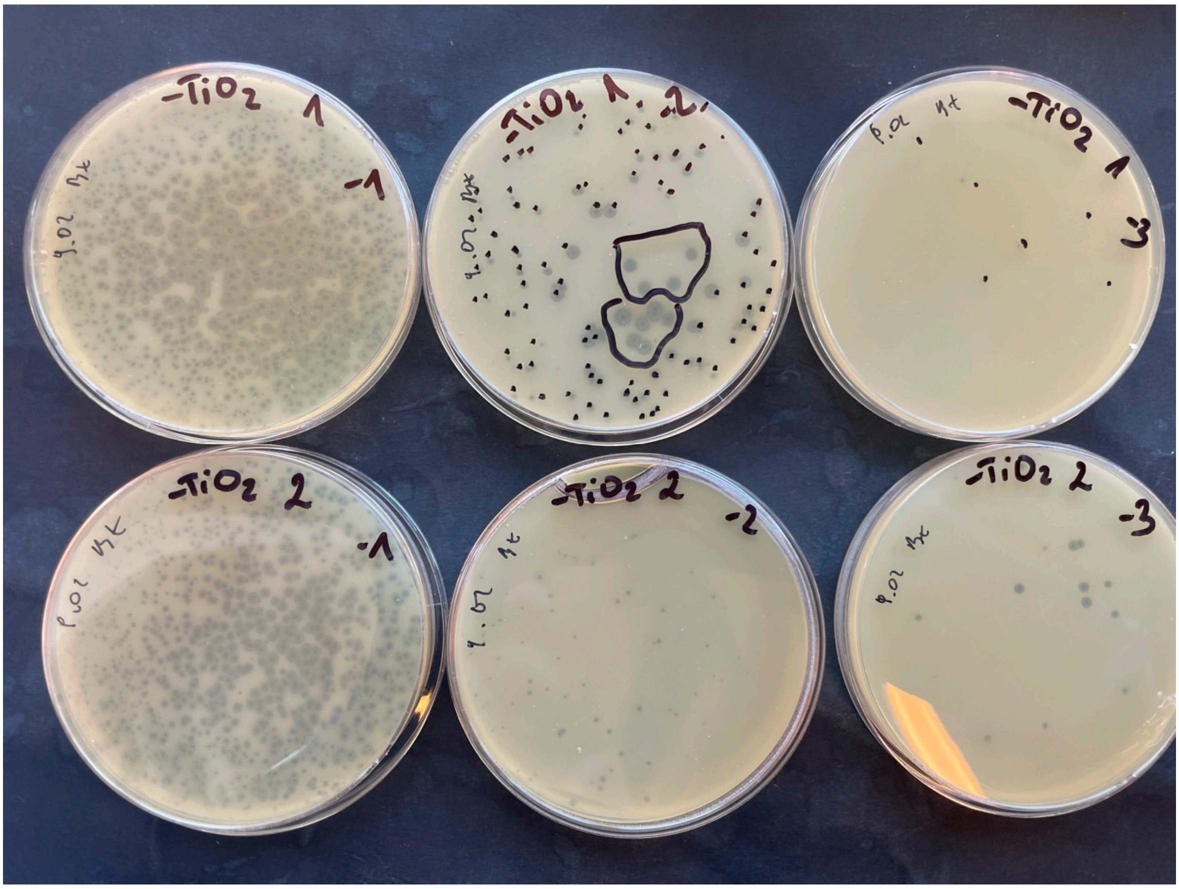
-TiO2 1 -1
-TiO2 1 -2
-TiO2 1 -3
-TiO2 2 -1
-TiO2 2 -2
-TiO2 2 -3

Figure S8.1. Representative double-agar-layer plaque plates from the first titration session (dilution series, 10^{-1}–10^{-3}), showing lysis plaques of TP-84 on lawns of *G. stearothermophilus* 10 StR for the input phage, the ground supports (± TiO_2) and the filter eluate.

Table S8.2. Repeat titration on subsequent days — four replicate platings of the 10^{-2} dilution (technical replicates used to obtain the mean titre). Values are plaques / PFU mL^{-1}.

Preparation	10^{-2}, replicate 1	10^{-2}, replicate 2	10^{-2}, replicate 3	10^{-2}, replicate 4
Start Sal 10^5	94 / 9.4×10^4	96 / 9.6×10^4	91 / 9.1×10^4	120 / 1.2×10^5
– TiO_2 1	98 / 9.8×10^4	116 / 1.7×10^5	79 / 7.9×10^4	105 / 1.0×10^5
– TiO_2 2	98 / 9.8×10^4	84 / 8.4×10^4	78 / 7.8×10^4	83 / 8.3×10^4
+ TiO_2 3	91 / 9.1×10^4	81 / 8.1×10^4	94 / 9.4×10^4	84 / 8.4×10^4
+ TiO_2 4	86 / 8.6×10^4	74 / 7.4×10^4	84 / 8.4×10^4	58 / 5.8×10^4
F (filter)	37 / 3.7×10^4	46 / 4.6×10^4	35 / 3.5×10^4	31 / 3.1×10^4

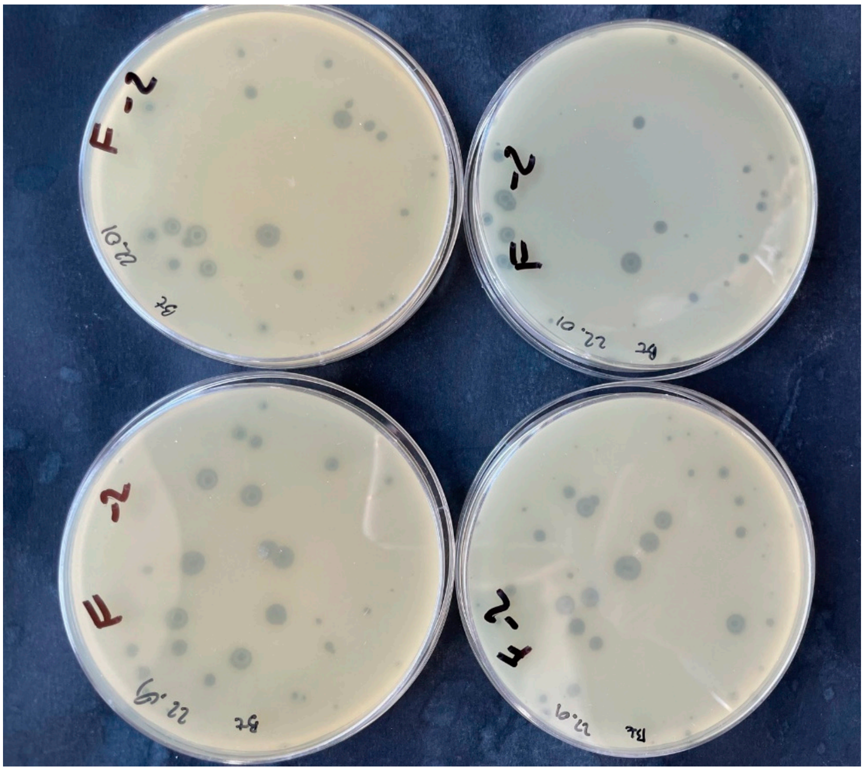

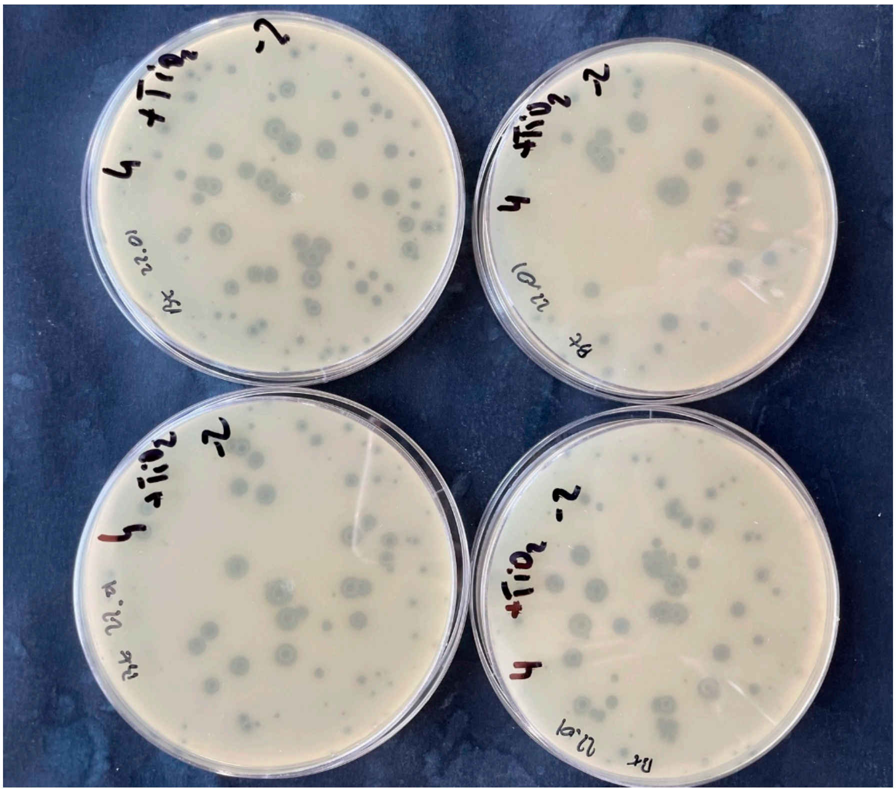
4 +TiO2 -2

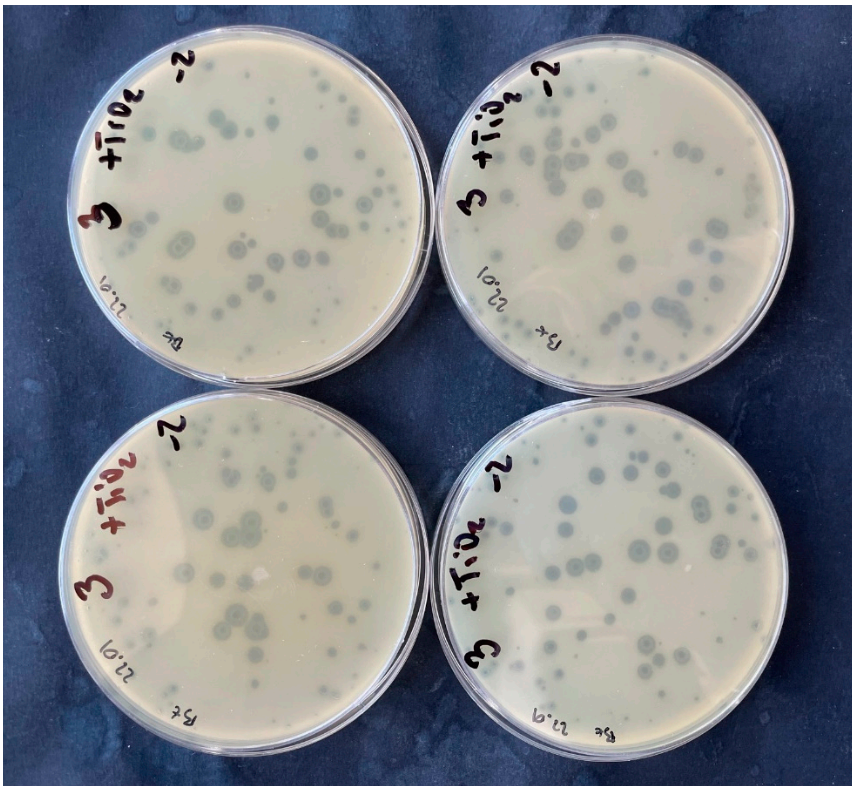
3 +TiO2 -2

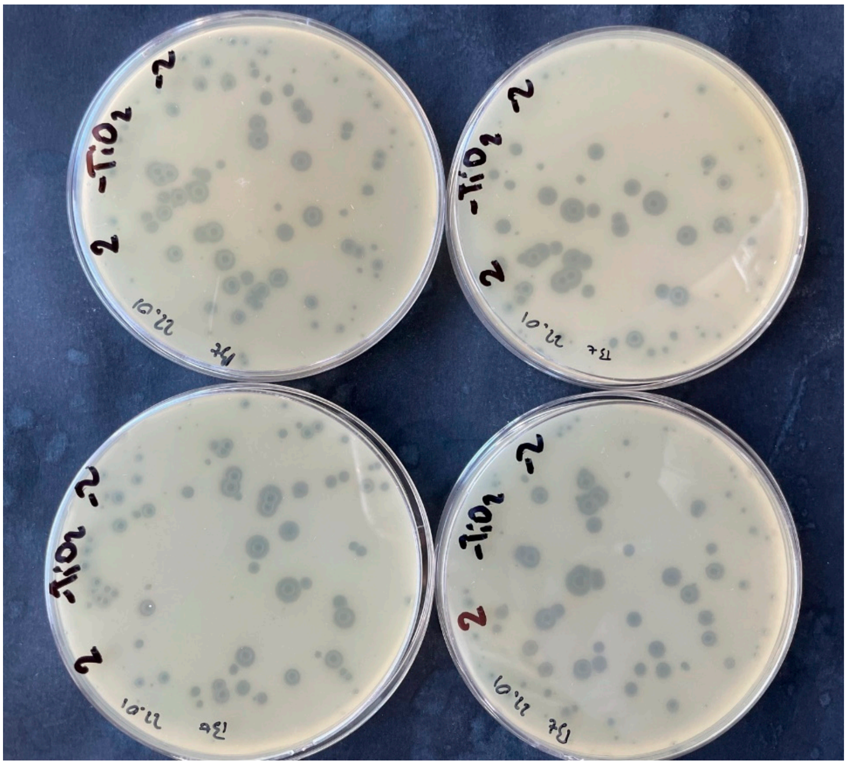
2 -TiO2 -2
2 -TiO2 -2
2 -TiO2 -2
2 -TiO2 -2
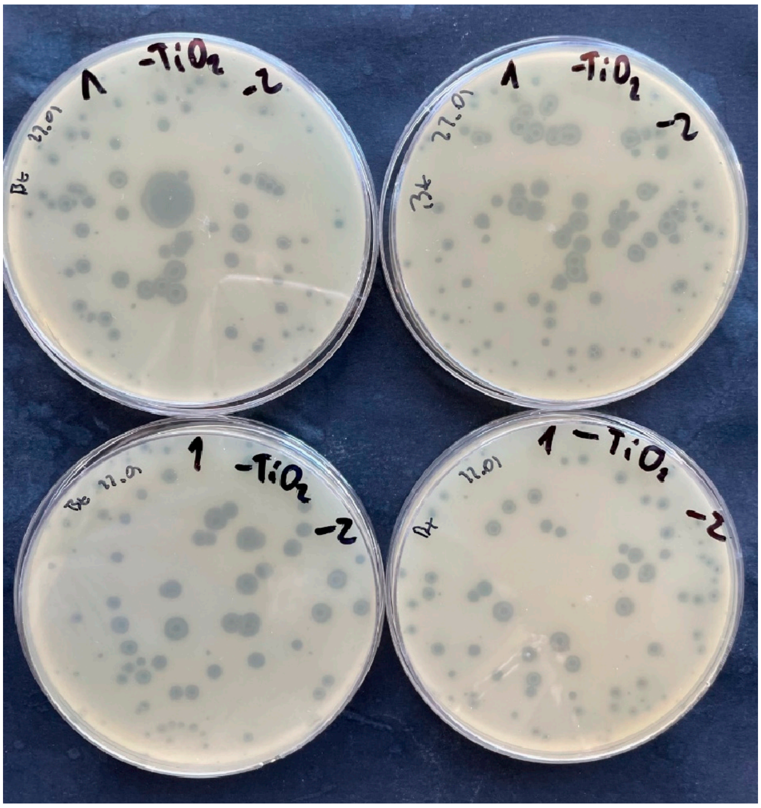
1 -TiO2 -2
1 -TiO2 -2
1 -TiO2 -2
1 -TiO2 -2

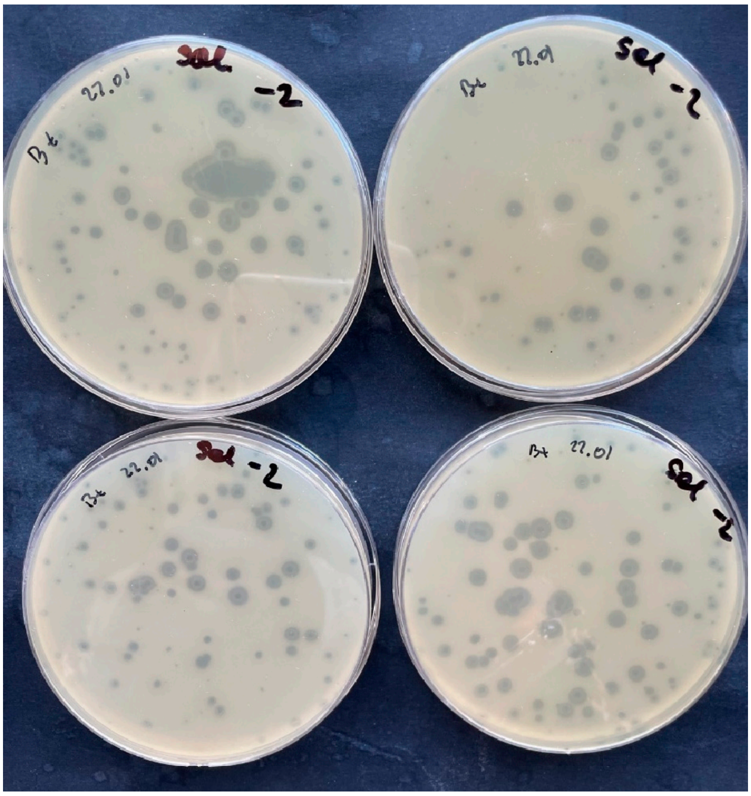

Figure S8.2. Plaque plates from the quadruplicate 10^{-2} titrations performed on subsequent days, from which the mean titre of each preparation was determined.
